# Diagnosis and preoperative evaluation of vena cava mass by endoscopic ultrasound: “hunting” for the overlooked lesions

**DOI:** 10.1055/a-2192-0576

**Published:** 2023-11-24

**Authors:** JinYu Wu, JiaYun Xu, ZengMin Zong, CuiWei Zhang, Huan Xu, Lei Shi

**Affiliations:** 1 556508 Department of Gastroenterology, The Affiliated Hospital of Southwest Medical University, Luzhou, China; 2 507824 Pathology, Southwest Medical University School of Clinical Medical Sciences, Luzhou, China

A 53-year-old woman was admitted to our hospital with right-sided lower abdominal pain lasting for 1 year. Her laboratory tests were unremarkable. Computed tomography (CT) and CT angiography revealed a 4.7 × 4.0 × 5.4 cm mass along the posterior head of the pancreas and invading the inferior vena cava (IVC) ( Fig. 1 , Video 1 ). Endoscopic ultrasound (EUS) found a solid hypoechoic lesion separate from the pancreas, which was growing transmurally and partially protruding outside the IVC wall and encroaching upon the right renal and hepatic veins. For a definitive diagnosis, we performed EUS-guided fine-needle aspiration (EUS-FNA) of the tissue mass through the duodenum, using a 22-gauge needle ( Fig. 2 ).

**Fig. 1:**
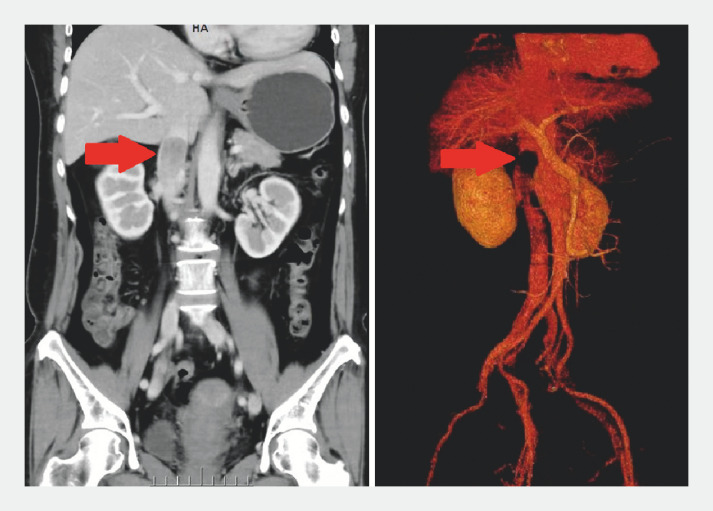
Computed tomography (CT; left) and CT angiography (right) revealed a 4.7 × 4.0 × 5.4 cm mass (arrow) along the posterior head of the pancreas and invading the inferior vena cava.

**Fig. 2:**
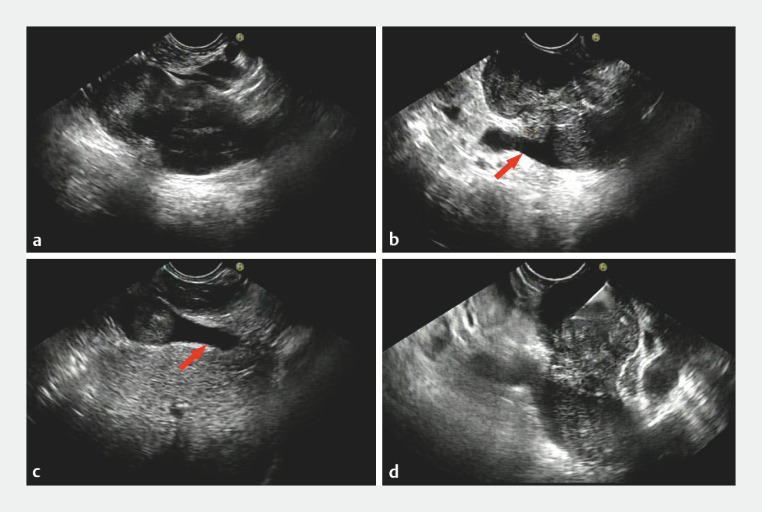
Endoscopic ultrasound (EUS) images. **a** Inferior vena cava. **b** Right renal vein (arrow). **c** Hepatic vein (arrow). **d** EUS-guided fine-needle aspiration of the tissue mass through the duodenum, using a 22-gauge needle.

Diagnosis and preoperative evaluation of vena cava mass by endoscopic ultrasound.Video 1

The pathological examination found that the mass was composed of spindle cells, and immunohistochemistry showed that it was positive for vimentin, desmin, SMA, H-caldesmon, and Ki67 (+, 40%), and negative for CD34, CD117, DOG1, S100, SOX11, MDM2, CDK4, and p16, indicating the diagnosis of leiomyosarcoma ( Fig. 3 ). The patient was referred to surgeons for further management.

**Fig. 3:**
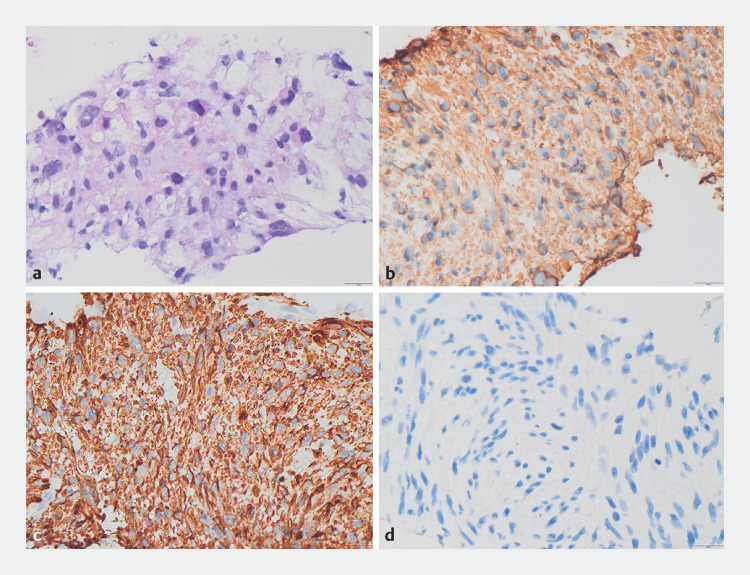
Histopathology of tissue mass samples (×400). **a** Hematoxylin and eosin staining showed spindle cells. **b–d** Immunohistochemical staining showed positivity for desmin ( **b** ) and SMA ( **c** ), and negativity for CD34 ( **d** ).

Leiomyosarcoma of the IVC is a rare cause of unexplained abdominal pain in patients 1 . Biopsy is generally considered challenging due to its deep location and risk of bleeding from the IVC, and preoperative misdiagnosis and diagnostic delays before surgery are frequent, which means that many patients are initially treated inappropriately 2 3 . Different surgical resection techniques and approaches are dependent on the level of IVC involvement, the tumor extension, and the presence or absence of collateral veins 4 . In our case, we demonstrated that EUS-FNA may be a simple, safe, and minimally invasive diagnostic method for a vena cava mass. Moreover, EUS could detect invasion of the right renal and hepatic veins more accurately than CT angiography, which is helpful when making a precise plan for surgery.

Endoscopy_UCTN_Code_CCL_1AF_2AZ_3AB

## Notes

Endoscopy E-Videos https://eref.thieme.de/e-videos
*E-Videos* is an open access online section of the journal *Endoscopy* , reporting on interesting cases and new techniques in gastroenterological endoscopy. All papers include a high-quality video and are published with a Creative Commons CC-BY license. Endoscopy E-Videos qualify for HINARI discounts and waivers and eligibility is automatically checked during the submission process. We grant 100% waivers to articles whose corresponding authors are based in Group A countries and 50% waivers to those who are based in Group B countries as classified by Research4Life (see: https://www.research4life.org/access/eligibility/ ). This section has its own submission website at https://mc.manuscriptcentral.com/e-videos .
